# Prognostic factors in carpal tunnel syndrome treated with 5% dextrose perineural injection: A retrospective study

**DOI:** 10.7150/ijms.56142

**Published:** 2021-03-03

**Authors:** Tsung-Yen Ho, Si-Ru Chen, Tsung-Ying Li, Chun-Yi Li, King Hei Stanley Lam, Liang-Cheng Chen, Yung-Tsan Wu MD

**Affiliations:** 1 Department of Physical Medicine and Rehabilitation, Tri-Service General Hospital, School of Medicine, National Defense Medical Center, No. 325, Sec. 2, Cheng-Kung Road, Neihu District, Taipei, Taiwan, Republic of China.; 2 Department of Physical Medicine and Rehabilitation, Taichung Armed Forces General Hospital, No. 348, Sec. 2, Chungshan Road, Taiping District, Taichung City, Taiwan, Republic of China.; 3 Integrated Pain Management Center, Tri-Service General Hospital, School of Medicine, National Defense Medical Center, No. 325, Sec. 2, Cheng-Kung Road, Neihu District, Taipei, Taiwan, Republic of China.; 4 The Hong Kong Institute of Musculoskeletal Medicine, Hong Kong.; 5 Department of Family Medicine, The Chinese University of Hong Kong, Hong Kong.; 6 Department of Family Medicine, The University of Hong Kong, Hong Kong.

**Keywords:** Carpal tunnel syndrome, Glucose, Prognostic factor

## Abstract

**Background:** Perineural injection therapy with 5% dextrose water (D5W) is a potential and innovative treatment with long-term efficacy for carpal tunnel syndrome (CTS). However, the prognostic factors of this management are lacking; hence, the aim of this retrospective study was to identify the prognostic factors of D5W perineural injection therapy for mild-to-moderate CTS.

**Methods:** A total of 52 patients (52 wrists) diagnosed with mild-to-moderate CTS and treated with a single ultrasound-guided 5cc D5W perineural injection were retrospectively reviewed. Patient-reported injection outcomes (visual analog scale, VAS) at 6 months post-injection were categorized into two groups; (1) Good outcome, when symptom relief ≥50% compared to pre-injection and (2) Poor outcome, when symptom relief < 50% compared to pre-injection. Significant variables between groups were entered into a binary logistic regression with forward stepwise regression to determine the prognostic factors for these outcomes.

**Results:** The treatment outcome was significantly related to body height and sensory nerve conduction velocity (SNCV) (159.1 ± 1.0 vs. 155.0 ± 1.8, *p*=0.04; 33.6 ± 0.8 vs. 28.3 ± 1.2, *p*=0.001, good vs. poor outcomes). However, only SNCV remained significantly correlated with the outcomes after conducting stepwise logistic regression (ORs: 1.201; 95% CI 1.05-1.38; *p*=0.01).

**Conclusions:** SNCV was found to be a significant prognostic factor of treatment outcome for patients with mild-to-moderate CTS 6 months after a D5W perineural injection.

## Introduction

Carpal tunnel syndrome (CTS), the most well-known peripheral neuropathy, is caused by the entrapment of the median nerve (MN) in the carpal tunnel, which leads to high intracarpal tunnel pressure. The increased pressure results in ischemia of the nerve, proceeding to paresthesia, pain and even muscle weakness and atrophy. Although there is no standard guideline for CTS treatment, mild-to-moderate CTS cases are initially given conservative treatment, whereas surgery is reserved for severe cases or patients who have had a poor response to conservative treatment. Conversely, current conservative treatments only have limited therapeutic benefits and short-term efficacy. A recent systematic review revealed that 57% to 66% of patients who have undergone conservative CTS treatment eventually receive surgery after 1-3 years 1.

Perineural injection therapy (PIT) with 5% dextrose water (D5W) is a potential and innovative treatment with long-term effects for CTS, based on the results from the latest series of high-quality clinical trials 2-5. Further, it has been recommended as an alternative local treatment as it does not have the side effects of corticosteroids, even though its benefits have only been shown by two research teams in single country and the mechanism of action is not completely understood 6. Hence, it is essential to identify the prognostic factors of PIT with D5W for CTS because this information aids decision making and informs patient expectations. Nonetheless, the associated information has yet to be investigated. Therefore, the aim of this retrospective study was to recognize the prognostic factors of treatment outcome after PIT with D5W for mild-to-moderate CTS.

## Material and methods

### Study design

This retrospective study was approved by the institutional review board of our hospital (No. 1-108-05-039) and the informed consent was waived. A total of 52 patients (60 wrists) who had participated in our previous clinical trial and had received single ultrasound-guided PIT with D5W after a diagnosis of mild-to-moderate CTS between May 2016 to April 2018 were secondarily analyzed 2, 3. Both wrists were allocated to the same group if the patient was diagnosed with bilateral CTS. For patients receiving bilateral injections, data from the more symptomatic side were recorded for analysis. All the patients completed a 6 month follow-up post-injection and all therapy regarding CTS treatments, except acetaminophen (500 mg, up to 4 g per day), were stopped 2 weeks prior to the injection and for 6 months after the injection 2, 3. Records of baseline characteristics and demographic data, symptom duration, electrophysiological study, and ultrasonographic cross-sectional area (CSA) of MN measured at baseline prior to injection were reviewed. The body weight and height were measured using a standard measuring instruments. The severity of symptoms and disability were evaluated through the use of the visual analog scale (VAS) and Boston Carpal Tunnel Syndrome Questionnaire (BCTQ), where a higher score indicated greater severity 2-4.

### Inclusion and exclusion criteria

Patients aged > 20 years with single or bilateral mild-to moderate CTS based on symptoms persisting at least over 3 months with confirmed diagnosis from the electrophysiological study were enrolled 2, 3. Definition of clinical presentations were: (1) Paresthesia/ dysesthesia with weak and clumsy hand. Symptoms exacerbated by repeated wrist use or falling asleep and symptoms subsiding after postural adjustment or hand shaking, (2) Numbness due to sensory impairment in MN innervated territory, (3) Thenar muscle weakness ± atrophy, and (4) Positive Phalen's test ± Tinel's sign. CTS was diagnosed if patients fit criteria 1 as well as one or more of criteria 2, 3, or 4 2-4. Exclusion criteria included previous history of wrist surgery, polyneuropathy, brachial plexopathy, or thoracic outlet syndrome, systematic infection, pregnancy or previous steroid injection for CTS 6 months prior to PIT with D5W.

### Electrophysiological study and CTS grading

All examinations were conducted by the same physician in the same room with the temperature maintained at 25 °C. Skin temperatures on the hand and wrist were maintained between 32.0 °C and 34.0 °C. The antidromic sensory nerve conduction velocity (SNCV) was recorded with the stimulator 14 cm proximal to the active electrode at the 2^nd^ interphalangeal joint and distal motor latency (DML) was measured with the stimulator 8 cm proximal to the active electrode on the abductor pollicis brevis muscle. The diagnosis and grading of the electrophysiological study were as follows. A cutoff value for normal MN's SNCV was < 3.6 ms and for normal MN's DML was < 4.3 ms. The grading of CTS by the electrophysiological study were classified as (1) Mild: abnormal SNCV with a normal DML, (2) Moderate: an abnormal SNCV and DML, and (3) Severe: SNCV was absent and DML was abnormal 7-9. The mean data of three repeated tests were used for analysis.

### Cross-sectional area of nerve

The CSA of the MN was assessed by means of electronic calipers at the proximal inlet of the carpal tunnel (scaphoid-pisiform level) using ultrasonography as previously described 2-4. The mean data of three repeated tests were used for analysis.

### Ultrasound-guided PIT with D5W

The same physician conducted an ultrasound-guided injection using ultrasonography (MyLab™25Gold, Esaote, Genova, Italy) as previously described 2, 3. All patients underwent a single PIT with 5 cc D5W under a double-blind design 2, 3. In summary, at the scaphoid-pisiform level, the MN was identified at the proximal inlet of the carpal tunnel. Using the in-plane short-axis ulnar approach, 2 cc D5W was injected to dissect the MN from the subsynovial connective tissue. Then, the residual 3 cc D5W was injected to hydrodissect the MN from the flexor retinaculum.

### Outcome measurement

VAS, was used to record the severity of pain or paresthesia, with the score ranging from 10 (remarkable pain) to 0 (no pain), 1 week before evaluation 2, 3. The patient-reported injection outcome at 6 months post-injection was categorized into (1) Good outcome, when symptom relief (VAS scores) ≥50% compared to pre-injection and (2) Poor outcome, when symptom relief (VAS scores) < 50% compared to pre-injection 4.

### Patient and public involvement

There was no patient or public involvement in this study.

### Statistical analysis

All collected data were statistically analyzed by using SPSS statistics version 22. Mann-Whitney U test and Chi-square tests or Fishers exact tests were used to analyze continuous and categorical demographic data, respectively. The noteworthy variables (*p* < 0.05) between groups were moved into a binary logistic regression with forward stepwise regression to define the prognostic factors for the outcome. Statistical significance was set at *p* < 0.05.

## Results

The mean age and symptom duration were 58.2 ± 1.7 years and 44.3 ± 5.9 months respectively of all 52 patients (52 wrists). There was 26.9% (14/52) patients diagnosed as mild grade and 73.1% as moderate grade CTS. A total of 39 patients (75%) rated a good outcome and 13 patients (25%) scored a poor outcome 6 months after undergoing PIT with D5W. The body height (BH) was significantly higher in the good outcome group than in the poor outcome group (159.1 ± 1.0 vs. 155.0 ± 1.8, p=0.04). In addition, the SNCV was significantly higher in the good outcome group than the poor outcome group (33.6 ± 0.8 vs. 28.3 ± 1.2, p=0.001). Although DML and CSA were greater, and the symptom duration, moderate grade classification, VAS, and BCTQ scores appeared to be higher in the poor outcome group than in the good outcome group, none of these measures reached significance (Table 1).

The results from the forward stepwise logistic regression for prognostic factor of good and poor outcome are shown in Table 2. Two factors (BH and SNCV) significantly correlated with the outcome. However, only SNCV remained significantly correlated with the outcome when the stepwise logistic regression was conducted (ORs: 1.201; 95% CI 1.05-1.38; *p*=0.01). Therefore, SNCV can be considered as the only significant prognostic factor of the treatment outcome.

## Discussion

The presented study was the first to investigate prognostic factors for mild-to-moderate CTS treated with D5W perineural injection. The results revealed BH and SNCV were all associated with the outcome; however, only SNCV was found to be a prognostic factor of the outcome. Likewise, severe SNCVs could be used to significantly predict poor outcomes at 6 months post-injection and patients with severe SNCV had a 1.201 times greater risk of poor outcome regardless of comorbidities, symptom duration and severity geades.

In 2017, Wu et al. 3 published a randomized double-blind clinical trial to show that the beneficial effects (i.e. reduced pain, disability, CSA of MN and electrophysiological improvement) after a single ultrasound-guided PIT with 5cc D5W in patients with mild-to-moderate CTS lasted at least 6 months. Moreover, they showed that this novel injection was superior to the corticosteroid injection at 4 and 6 months post-injection as the beneficial effects after PIT with D5W were more pronounced than after the corticosteroid injection 2. Li et al. 4 retrospectively traced 185 patients with varied grades of CTS who received ultrasound-guided PIT with 10cc D5W (mean 2.2 times) at least 1-year post-injection (mean 1-3 years follow-up). The results showed that 88.6% patients had effective outcomes (symptom relief ≥50% compared to pre-injection) and 62.7% patients rated an excellent outcome (symptom relief ≥70%). Moreover, only 2 patients received surgery in their study. Recently, Lin et al. 5 verified the volume-dependent effects of PIT with D5W which higher volume provided better efficacy of symptom and functional improvement for CTS. Hence, PIT with D5W could significantly increase the success rate and decrease the surgical rate compared to current conservative treatment, in which 57% to 66% of patients ultimately receive surgery after 1 to 3 years due to the failure of conservative treatments 1. The reason why the effect of D5W injection for CTS has not yet been replicated elsewhere by another research group in other country maybe due to different medical systems, resources and cultures in different conuntries. Actually, this innovative treatment was recently confirmed to be effective for CTS since 2017 and there are some ongoing trials from other research groups now. Hence, there will be more research to verify its effect in the future.

In the retrospective study by Li et al. 4, the treatment outcome was only significantly related with severity grade of CTS, where the more severe grades have a significantly higher incidence of a poor outcome compared to mild-to-moderate grades after treatment. Nevertheless, no electrophysiological data were recorded in their study although the CSA of MN was used for analysis. Although our results have shown a larger CSA in the poor outcome group, CSA was not associated with the treatment outcome, which is comparable to the findings from the study by Li et al. Moreover, the severity grades demonstrated no association with the treatment outcome in our study although more moderate grades and higher VAS and BCTQ scores were observed in the poor outcome group; however, these findings were not significant (all *p* > 0.05). The discrepancy of the aforementioned results may arise from differences in injection technique, injection volume, number of injections and severity of grades.

Although electrophysiological study has been recommended as a standard diagnostic tool for CTS in the past decades, studies have shown its limitations because of varied sensitivity (56 to 85%) and specificity (94 to 96%) 10, 11. Further, it has been reported that the inconsistent relationships between the electrophysiological study and symptom severity for CTS 12. The high intracarpal tunnel pressure could damage nerve microcirculation inducing histologic changes including ischemia, impaired nerve conduction, MN dynamics decrement with adhesion, increased vascular permeability of MN and interrupted axoplasmic flow leading to focal nerve swelling proximal to the compression site 13, 14. Moreover, the fibrotic scar may encircle the MN accompanying with atrophy of nerve in chronicitiy of nerve entrapment 14. Hence, the ultrasonographic measurement has been used progressively in CTS diagnosis recently and the most used diagnostic criteria for CTS was the CSA of MN at the inlet of carpal tunnel, having high sensitivity (89%) and specificity (83%) 15, 16. In addition, most published studies established the relationship between CSA and symptom scores 16, 17, although, a few studies revealed no such association 18. However, when the MN is physiologically damaged, the histological evaluation by using ultrasonography may be normal in the early stage. Therefore, the electrophysiological findings maybe more correlated with symptom, in comparison to ultrasonographic measurements in early stages 18. This may explain why no significant correlations between ultrasonographic findings and symptom in some literature and our study. Although CSA was higher in the poor outcome group, varied symptom duration in our recruited patients may underestimate the association between CSA and outcome. Therefore, it would be valuable for further studies to separate patients regarding their symptom duration and then evaluate prognostic factors of treatment outcomes.

Electrophysiological study is one of the most reported prognostic factors in CTS and severe datas implied more demyelination or conduction block of MN. Gelberman et al. 19 demonstrated that prolonged DML and absent sensory nerve action potentials were correlated with a poor outcome after corticosteroid injection. Visser et al. 20 stated that only severity of electrodiagnostic results was a predictor of outcome after corticosteroid injections and a mild grade has a better outcome. Zwolińska et al. 21 reported that worse primary SNCV and DML are prognostic factors of a less favorable outcome after conservative treatment such as whirlpool massage, sonotherapy, and glide exercise. Moreover, CTS patients with better DML and sensory latencies were good candidates for wrist splinting 22, and failure of wrist splinting and corticosteroid injections could be predicted by long motor and sensory distal latency 23. Atroshi et al. 24 showed that better DML was the prognostic factor for dissatisfaction after surgery. Bland et al. 25 revealed that the electrophysiological study was a strong predictor of outcomes and that moderate grades had an improved outcome compared to those with either very severe or normal grades after surgical decompression. Dennerlein et al. 26 reported that prolonged preoperative DML and sensory latencies were associated with less symptom and functional limitations after surgery; however, the outcome could be predicted only by the DML. In contrast, some studies have found that pre-treatment results from the electrophysiological study did not correlate with the CTS outcome after surgery 27, 28 or corticosteroid injection 29. The discrepancy of the predictive value of the electrophysiological study from aforementioned studies echo current treatment recommendations that mild-to-moderate CTS cases are given conservative treatment, whereas surgery is reserved for severe cases or in patients who have a poor response to conservative treatments. Our results demonstrated that SNCV is an independent prognostic factor of outcome, where a worse SNCV score predicts poor outcomes for patients after the D5W injection. This finding was in line with previous research 20-23 and this information can be valuable for clinical practice of decision making and informs patient expectations.

The CSA of MN has previously been reported as a predictor for outcomes for CTS. Hui et al. 30 revealed that swollen MNs responded more satisfactorily to a corticosteroid injection over a 20-week period. Chung et al. 31 also reported that CSA of MN is a valuable predictor after corticosteroid injections at 4 weeks post-injection, where a larger CSA predicts a good outcome. Conversely, Meys et al. 29 found that smaller CSA of MN at onset was associated with a more successful outcome 1-year post-corticosteroid injection. Naranjo et al. 32 reported that the CSA of MN is a better predictor of outcome than the electrophysiological study, where larger CSA was predictive of good outcomes after surgical release. Mondelli et al. 33 showed that smaller initial CSA was predictive of better surgical outcomes. In contrast, some studies have shown that CSA was not predictive of outcomes after conservative management 34 or surgery 35. Although our results showed smaller CSA in the good outcome group at 6 months post-injection, the difference between the groups was not significant. The discrepancy of the associated outcome between CSA with corticosteroid or D5W injections may result from different pharmacological effects and follow-up duration. Corticosteroid injections could be more beneficial, as a result of its strong anti-inflammatory effects, for larger CSA (more swollen nerves) in the initial months after treatment. This is supported by the Cochrane review which suggested it only has short-term efficacy (i.e. one month) 36. Despite the anti-inflammatory properties of corticosteroids, D5W has been proposed to be more effective as it has possible anti-neurogenic inflammatory and regenerative effects 2. The D5W injection was proved to be more effective than corticosteroid injections at 4 and 6 months post-injection 2, hence, the smaller CSA of MN with longer follow-up duration was expected to be associated with good outcomes after D5W injections. However, the relatively small sample size with varied standard error in the control group may underestimate the association between CSA and outcome. Further study with a larger sample size and histological research is needed to explore the true mechanism of D5W.

Finally, a high BCTQ score indicates more severe symptoms and functional impairment and are the poor prognostic factors of CTS with conservative management 1 and surgery 37. In contrast, another study revealed that a low BCTQ score predicted minor improvement after surgery 38. A study with a design similar to ours showed slightly high initial BCTQ-severity but low BCTQ-function score in a good outcome group post-steroid injection, with only BCTQ-function showing statistical significance 39. Although relatively low BCTQ scores (severity and function) were observed for the good outcome in our study, the severity grades in both groups were similar. Hence, this could have resulted in the non-association between BCTQ scores and the outcome in our study. Moreover, the discrepancy between our study results and those of previous studies may also arise from the difference in inclusion/exclusion criteria, follow-up duration, and intervention methods.

There are a few limitations in our study. First, including detailed clinical data (e.g. Tinel sign, Phalen test or thenar muscle atrophy) would provide a more comprehensive analysis. Second, the single national studies of D5W injection for CTS may produce a population and geographic bias; therefore, other national research was expected in the future. Moreover, it would be more comprehensive to assess the satisfaction and quality of lives at the same time apart from only objective symptom scores. Finally, our study did not investigate prognostic factors of short-term outcomes (i.e. 3-month) and the association between symptoms and electrophysiological and CSA results, hence, future investigation into this would be beneficial.

In conclusion, this study found that severe SNCV had a 1.201 times greater risk of predicting poor outcome at 6 months post-injection with D5W for patients with mild-to-moderate CTS. However, future larger and more other national data sets should be investigated to support the current research results.

## Figures and Tables

**Table 1:** Baseline characteristics and comparison of demographic data of the study population

	All patients (n=52 wrists)	Good outcome (n=39 wrists)	Poor outcome (n=13 wrists)	^a^*p* value
**Gender, n (%)**				0.420
Female	43 (82.7)	31 (79.5)	12 (92.3)	
Male	9 (17.3)	8 (20.5)	1 (7.7)	
Age (year) ± SE (range)	58.2 ± 1.7 (31-84)	58.3 ± 2.0 (31-84)	58.1 ± 3.1 (40-80)	0.775
BH (cm) ± SE (range)	158.1 ± 0.9 (147-176)	159.1 ± 1.0 (147-176)	155.0 ± 1.8 (147-170)	0.040
BW (kg) ± SE (range)	66.9 ± 1.6 (44-100)	68.2 ± 1.8 (44-100)	63.0 ± 3.0 (44-80)	0.155
DM (%)	6 (11.5)	5 (12.8)	1 (7.7)	0.616
Hypertension (%)	25 (48.1)	19 (48.7)	6 (46.2)	0.873
**Handedness, n (%)**				1.000
Right	52 (100)	39 (100)	13 (100)	
Left	0 (0)	0 (0)	0 (0)	
**Lesion site, n (%)**				0.817
Left	21 (40.4)	16 (41.0)	5 (38.5)	
Right	31 (59.6)	23 (59.0)	8 (61.5)	
Duration (month) ± SE (range)	44.3 ± 5.9 (3-180)	43.5 ± 6.8 (3-180)	46.5 ± 12.3 (6-120)	0.992
**Padua classification (%)**				0.718
Mild	14 (26.9)	11 (28.2)	3 (23.1)	
Moderate	38 (73.1)	28 (71.8)	10 (76.9)	
VAS (SE)	6.5 ± 0.2	6.5 ± 0.2	6.6 ± 0.5	0.958
BCTQs (SE)	29.2 ± 0.9	28.9 ± 1.1	30.4 ± 1.9	0.409
BCTQf (SE)	21.2 ± 0.7	21.2 ± 0.8	21.5 ± 1.2	0.907
SNCV (m/s) (SE)	32.3 ± 0.7	33.6 ± 0.8	28.3 ± 1.2	0.001
DML (ms) (SE)	5.0 ± 0.2	4.8 ± 0.2	5.6 ± 0.4	0.203
CSA (mm^2^) (SE)	12.5 ± 0.3	12.5 ± 0.3	12.8 ± 0.5	0.799

SE, standard error; BH, body height; BW, body weight; DM, diabetes mellitus; VAS, Visual Analog Scale; BCTQ, Boston Carpal Tunnel Syndrome Questionnaire (s = severity, f = function); SNCV, sensory nerve conduction velocity; DML, distal motor latency; CSA, cross-sectional area. ^a^Mann-Whitney U test, Chi-square test or Fishers exact test.

**Table 2:** Stepwise logistic regression analysis of the study population and potential factors affecting outcome

Variable	Good outcome (n=39 wrists)	Poor outcome (n=13 wrists)	*p* value	Stepwise logistic regression		
				Odds ratio	95% CI	*p* value
SNCV (m/s) (SE)	33.6 ± 0.8	28.3 ± 1.2	0.001**	1.201	(1.05-1.38)	0.010**
BH (cm) (SE)	159.1 ± 1.0	155.0 ± 1.8	0.040*	1.102	(0.97-1.26)	0.151

SNCV, sensory nerve conduction velocity; SE, standard error; BH, body height. Mann-Whitney U test was used to compare the difference between two groups and significant variables was evaluated by binary logistic regression with forward stepwise; *p≤0.05, **p≤0.01.

## References

[1] Burton CL, Chesterton LS, Chen Y, van der Windt DA (2016). Clinical Course and Prognostic Factors in Conservatively Managed Carpal Tunnel Syndrome: A Systematic Review. Arch Phys Med Rehabil.

[2] Wu YT, Ke MJ, Ho TY, Li TY, Shen YP, Chen LC (2018). Randomized double-blinded clinical trial of 5% dextrose versus triamcinolone injection for carpal tunnel syndrome patients. Ann Neurol.

[3] Wu YT, Ho TY, Chou YC, Ke MJ, Li TY, Tsai CK (2017). Six-month Efficacy of Perineural Dextrose for Carpal Tunnel Syndrome: A Prospective, Randomized, Double-Blind, Controlled Trial. Mayo Clin Proc.

[4] Li TY, Chen SR, Shen YP, Chang CY, Su YC, Chen LC (2020). Long-term outcome after perineural injection with 5% dextrose for carpal tunnel syndrome: a retrospective follow-up study. Rheumatology.

[5] Lin MT, Liao CL, Hsiao MY, Hsueh HW, Chao CC, Wu CH (2020). Volume Matters in Ultrasound-Guided Perineural Dextrose Injection for Carpal Tunnel Syndrome: A Randomized, Double-Blinded, Three-Arm Trial. Front pharmacol.

[6] Josephson SA (2018). Injection of 5% Dextrose for Carpal Tunnel Syndrome More Effective Than Corticosteroid Injection. In: Kasper D ed. Harrison's Online Updates. New York, NY: McGraw-Hill Education.

[7] Rossi S, Giannini F, Passero S, Paradiso C, Battistini N, Cioni R (1994). Sensory neural conduction of median nerve from digits and palm stimulation in carpal tunnel syndrome. Electroencephalogr Clin Neurophysiol.

[8] Padua L, Lo Monaco M, Valente EM, Tonali PA (1996). A useful electrophysiologic parameter for diagnosis of carpal tunnel syndrome. Muscle Nerve.

[9] Padua L, LoMonaco M, Gregori B, Valente EM, Padua R, Tonali P (1997). Neurophysiological classification and sensitivity in 500 carpal tunnel syndrome hands. Acta Neurol Scand.

[10] Witt JC, Hentz JG, Stevens JC (2004). Carpal tunnel syndrome with normal nerve conduction studies. Muscle Nerve.

[11] Jablecki CK, Andary MT, Floeter MK, Miller RG, Quartly CA, Vennix MJ (2002). Practice parameter: Electrodiagnostic studies in carpal tunnel syndrome. Report of the American Association of Electrodiagnostic Medicine, American Academy of Neurology, and the American Academy of Physical Medicine and Rehabilitation. Neurology.

[12] Chen LC, Ho CW, Sun CH, Lee JT, Li TY, Shih FM (2015). Ultrasound-Guided Pulsed Radiofrequency for Carpal Tunnel Syndrome: A Single-Blinded Randomized Controlled Study. PLoS One.

[13] Uchiyama S, Itsubo T, Nakamura K, Kato H, Yasutomi T, Momose T (2010). Current concepts of carpal tunnel syndrome: pathophysiology, treatment, and evaluation. J Orthop Sci.

[14] Chang KV, Wu WT, Ozcakar L (2020). Ultrasound imaging and guidance in peripheral nerve entrapment: hydrodissection highlighted. Pain Manag.

[15] Wong SM, Griffith JF, Hui AC, Tang A, Wong KS (2002). Discriminatory sonographic criteria for the diagnosis of carpal tunnel syndrome. Arthritis Rheum.

[16] El Miedany YM, Aty SA, Ashour S (2004). Ultrasonography versus nerve conduction study in patients with carpal tunnel syndrome: substantive or complementary tests?. Rheumatology.

[17] Karadag YS, Karadag O, Cicekli E, Ozturk S, Kiraz S, Ozbakir S (2010). Severity of Carpal tunnel syndrome assessed with high frequency ultrasonography. Rheumatol Int.

[18] Kaymak B, Ozcakar L, Cetin A, Candan Cetin M, Akinci A, Hascelik Z (2008). A comparison of the benefits of sonography and electrophysiologic measurements as predictors of symptom severity and functional status in patients with carpal tunnel syndrome. Arch Phys Med Rehabil.

[19] Gelberman RH, Aronson D, Weisman MH (1980). Carpal-tunnel syndrome. Results of a prospective trial of steroid injection and splinting. J Bone Joint Surg Am.

[20] Visser LH, Ngo Q, Groeneweg SJ, Brekelmans G (2012). Long term effect of local corticosteroid injection for carpal tunnel syndrome: a relation with electrodiagnostic severity. Clin neurophysio.

[21] Zwolinska J, Kwolek A (2019). Factors determining the effectiveness of conservative treatment in patients with carpal tunnel syndrome. Int J Occup Med Environ Health.

[22] Nanno M, Kodera N, Tomori Y, Hagiwara Y, Takai S (2017). Electrophysiological Assessment for Splinting in the Treatment of Carpal Tunnel Syndrome. Neurol Med Chir (Tokyo).

[23] Stahl S, Yarnitsky D, Volpin G, Fried A (1996). Conservative therapy in carpal tunnel syndrome. Harefuah.

[24] Atroshi I, Johnsson R, Ornstein E (1998). Patient satisfaction and return to work after endoscopic carpal tunnel surgery. J Hand Surg Am.

[25] Bland JD (2001). Do nerve conduction studies predict the outcome of carpal tunnel decompression?. Muscle Nerve.

[26] Dennerlein JT, Soumekh FS, Fossel AH, Amick BC 3rd, Keller RB, Katz JN (2002). Longer distal motor latency predicts better outcomes of carpal tunnel release. J Occup Environ Med.

[27] Prick JJ, Blaauw G, Vredeveld JW, Oosterloo SJ (2003). Results of carpal tunnel release. Eur J Neurol.

[28] Longstaff L, Milner RH, O'Sullivan S, Fawcett P (2001). Carpal tunnel syndrome: the correlation between outcome, symptoms and nerve conduction study findings. J Hand Surg Br.

[29] Meys V, Thissen S, Rozeman S, Beekman R (2011). Prognostic factors in carpal tunnel syndrome treated with a corticosteroid injection. Muscle Nerve.

[30] Hui AC, Wong S, Leung CH, Tong P, Mok V, Poon D (2005). A randomized controlled trial of surgery vs steroid injection for carpal tunnel syndrome. Neurology.

[31] Chung SY, Kwak JM, Kang S, Son SH, Kim JD, Yoon JS (2018). Predictive Variables for Sonographically Guided Corticosteroid Injection in Mild-to-Moderate Carpal Tunnel Syndrome. Ann Rehabil Med.

[32] Naranjo A, Ojeda S, Arana V, Baeta P, Fernandez-Palacios J, Garcia-Duque O (2009). Usefulness of clinical findings, nerve conduction studies and ultrasonography to predict response to surgical release in idiopathic carpal tunnel syndrome. Clin Exp Rheumatol.

[33] Mondelli M, Filippou G, Aretini A, Frediani B, Reale F (2008). Ultrasonography before and after surgery in carpal tunnel syndrome and relationship with clinical and electrophysiological findings. A new outcome predictor?. Scand J Rheumatol.

[34] Marschall A, Ficjian A, Stradner MH, Husic R, Zauner D, Seel W (2016). The Value of Median Nerve Sonography as a Predictor for Short- and Long-Term Clinical Outcomes in Patients with Carpal Tunnel Syndrome: A Prospective Long-Term Follow-Up Study. PloS one.

[35] Bland JD, Rudolfer SM (2014). Ultrasound imaging of the median nerve as a prognostic factor for carpal tunnel decompression. Muscle Nerve.

[36] Marshall S, Tardif G, Ashworth N Local corticosteroid injection for carpal tunnel syndrome. Cochrane Database Syst Rev. 2007: CD001554.

[37] Bowman A, Rudolfer S, Weller P, Bland JD (2018). A prognostic model for the patient-reported outcome of surgical treatment of carpal tunnel syndrome. Muscle Nerve.

[38] Jansen M, Evers S, Slijper H, de Haas K, Smit X, Hovius S (2018). Predicting clinical outcome after surgical treatment in patients with carpal tunnel syndrome. J Hand Surg Am.

[39] Chung SY, Kwak JM, Kang S, Son S-H, Do Kim J, Yoon JS (2018). Predictive variables for sonographically guided corticosteroid injection in mild-to-moderate carpal tunnel syndrome. Ann Rehabil Med.

